# Association between circulating levels of miR-29 and postoperative neurological complications in acute type A aortic dissection patients

**DOI:** 10.3389/fcvm.2026.1636861

**Published:** 2026-02-27

**Authors:** Xiao-chai Lv, Yong Lin, Yan-ting Hou, Min-xia Xie, Liang-wan Chen

**Affiliations:** 1 Department of Cardiovascular Surgery, Fujian Medical University Union Hospital, Fuzhou, Fujian, China; 2 Key Laboratory of Cardio-Thoracic Surgery (Fujian Medical University), Fujian Province University, Fuzhou, Fujian, China; 3 Fujian Provincial Center for Cardiovascular Medicine, Fuzhou, Fujian, China

**Keywords:** acute type A aortic dissection, biomarker, deep hypothermic circulatory arrest, miR-29a-5p/miR-29b-3p, postoperative neurological complications, predictive nomogram

## Abstract

**Objectives:**

Postoperative neurological complications (PONC), which are associated with substantial morbidity and mortality, represent a prevalent clinical challenge following surgical repair of acute type A aortic dissection (AAD). This study aimed to identify novel biomarkers for the early diagnosis of PONC, facilitating timely clinical intervention.

**Methods:**

We established deep hypothermic circulatory arrest (DHCA) rat models, extracted total RNA from the hippocampus of rats (DHCA and control groups), performed microRNA (miRNA) sequencing, screened for differentially expressed genes (DEGs) between the two groups, and analysed their associated biological processes and pathways. A cohort of 95 patients with AAD was included in this study. Comprehensive clinical assessments and a standardized neuropsychological test battery were systematically conducted. Serum miR-29 levels were quantified via reverse transcription quantitative real-time polymerase chain reaction.

**Results:**

Transcriptomic profiling of the rat hippocampus under DHCA/cardiopulmonary bypass (CPB) revealed 31 differentially expressed miRNAs (FC > 1.5, *P* < 0.05), with miR-29a-5p and miR-29b-3p showing the most significant dysregulation. Functional enrichment analysis revealed that MAPK signalling and cellular junction pathways are involved in blood–brain barrier modulation. To translate these findings clinically, we analysed a cohort of 95 AAD patients. Compared with patients without PONC, those who developed PONC had significantly longer CPB duration [164.00 (137.00–193.00) vs. 140.00 (120.25–161.00) min; *P* = 0.012], higher preoperative interleukin-6 levels [106.60 (87.80–154.90) vs. 47.00 (35.45–71.73) pg/mL; *P* < 0.001], and altered miR-29 expression profiles. Multivariate analysis confirmed that preoperative miR-29b-3p (OR = 2.53, 95% CI 1.17–5.47) and postoperative miR-29a-5p (OR = 0.21, 95% CI 0.05–0.96) were independent predictors of PONC. The nomogram demonstrated robust discrimination (AUC = 0.867) and clinical utility (net benefit = 0.23), with 30-day survival analysis revealed an increased risk of mortality associated with miR-29b-3p (*P* = 0.041).

**Conclusions:**

This study identified dysregulated miR-29 as a key mechanism linked to PONC after CPB/DHCA and validated circulating miR-29b-3p as an independent predictor of PONC and mortality in AAD patients, providing a basis for early risk assessment.

## Introduction

Acute type A aortic dissection (AAD), a life-threatening vascular emergency, necessitates urgent surgical intervention to prevent fatal rupture (1). Despite advancements, postoperative neurological complications (PONCs) remain critical clinical challenges. Among these, cerebral dysfunction manifests as cognitive decline, memory impairment, and psychomotor deficits and significantly affects prognosis (2, 3). Current diagnostic limitations, compounded by individual variability in injury severity (4), underscore the urgent need for reliable biomarkers to enable early detection and risk stratification of postoperative neurological sequelae (5).

Among the database of outcome predictors after PONC, serum biomarkers are considered particularly valuable due to their clinical convenience. Emerging evidence indicates that systemic inflammation can compromise the blood–brain barrier (BBB), leading to endothelial dysfunction and facilitating the infiltration of peripheral immune cells and associated inflammatory mediators into brain tissue (6). Evidence suggests that inflammatory mediators, such as interleukin-6 (IL-6) and C-reactive protein (CRP), play important roles in the development of postoperative brain dysfunction (7). Additionally, serum levels of S100B protein and neuron-specific enolase (NSE) are widely used to evaluate disease severity and predict prognosis in hypoxic brain injury (8, 9). Although biomarkers such as S100B and NSE have been established for neuroprognostication, serum microRNA (miRNA) profiles in the context of AAD-induced cerebral injury remain uncharacterized.

miRNAs are a class of evolutionarily conserved small noncoding RNAs that are ubiquitously expressed the central nervous system, where they orchestrate critical neurobiological processes, including neuronal differentiation, synaptic development, and activity-dependent plasticity (10). The remarkable stability of circulating miRNAs in biological fluids, coupled with their tissue-specific expression patterns, has positioned these molecules at the forefront of biomarker discovery for neurological disorders (11). Emerging evidence has demonstrated consistent dysregulation of miRNA profiles in both peripheral circulation and cerebral tissue following various forms of neural injury (12). Notably, trauma-induced miRNAs exhibit temporal expression patterns that correlate with BBB integrity and neuroinflammatory responses (6). For instance, the expression of miR-132, a key regulator of synaptic plasticity, is significantly reduced in cerebrospinal fluid after brain injury and correlates with cognitive deficits in animal models (13). Similarly, Han et al. revealed the potential role of miR-124 in inflammation and its promise as both a future biomarker and a therapeutic target for neurodegenerative disorders (14). While these findings underscore the diagnostic potential of miRNA signatures in experimental settings, critical knowledge gaps persist regarding their clinical translatability. In particular, the predictive value of perioperative miRNA fluctuations for assessing neurological vulnerability and long-term functional outcomes in surgical patients remains to be systematically validated through multicenter longitudinal studies (15).

Emerging research has demonstrated the predominant cerebral enrichment of the miR-29 family, members of which critically modulates neurodevelopmental processes, including cortical maturation and neuronal process extension (16–18). Given the high incidence of PONC following AAD surgery and the well-documented involvement of the miR-29 family in regulating neuronal survival and ischemia‒reperfusion injury, we sought to investigate the potential association between miR-29 family and PONC in AAD.

## Materials and methods

### Experimental animals

All experimental procedures were conducted in accordance with the National Institutes of Health Guidelines for the Care and Use of Laboratory Animals and were approved by the Institutional Animal Care and Use Committee of Fujian Medical University. Nine-week-old male Sprague‒Dawley rats (weight range: 300–400 g) were housed under standard laboratory conditions (12 h light/dark cycle, 22 ± 1 °C, and 55 ± 5% humidity) with *ad libitum* access to food and water.

### Establishment of deep hypothermic circulatory arrest and a cardiopulmonary bypass animal model

**Figure 1:**
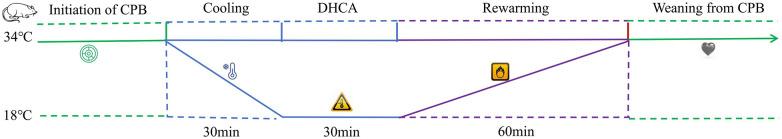
Flowchart of the establishment of a rat model of DHCA. DHCA, deep hypothermic circulatory arrest; CPB, cardiopulmonary bypass.

A rat model of cardiopulmonary bypass (CPB) with deep hypothermic circulatory arrest (DHCA) was established based on an adapted methodology (19). The detailed surgical and perfusion protocol proceeded was as follows:

1. Preoperative preparation and anaesthesia: After a 6-hour fast, the rats were weighed and anaesthetized. Surgical anaesthesia was then maintained by continuous inhalation of 3% sevoflurane in 100% oxygen throughout the surgical procedures, except for the circulatory arrest period itself.
2. Intubation and initial ventilation: Following supine positioning and fixation, tracheal intubation was performed using a 16-G catheter. The animal was connected to a rodent ventilator, which delivered oxygen with sevoflurane.
3. Monitoring and vascular access: Core temperature was monitored continuously via a rectal probe. For CPB, venous drainage was established by cannulating the external jugular vein with a silicone catheter. Arterial perfusion was achieved via the tail artery, and systemic arterial pressure was monitored via the left femoral artery. All vascular access points were secured by distal ligation and proximal fixation. The CPB circuit incorporated a membrane oxygenator (Xi'an Xijing Medical Supplies Co., Ltd.).
4. CPB and DHCA protocol: The animal was connected to the CPB circuit, which was integrated with a heater-cooler unit (Stockert S5, Maquet). The definitive DHCA protocol comprised five sequential phases (Figure 1): (1) CPB initiation: CPB commenced at a flow rate of 160–180 mL/kg/min and was maintained under normothermic conditions for 5 min. (2) Cooling phase: Active cooling via the CPB circuit was performed to decrease the rectal temperature to a target of 18 °C over 30 min. (3) Circulatory arrest: Once the target temperature was reached, full circulatory arrest was initiated and maintained for 30 min. Mechanical ventilation was paused during this period. (4) Rewarming and reperfusion: CPB flow and mechanical ventilation were resumed. Slow rewarming to a rectal temperature of 34 °C was conducted for approximately 60 min. (5) Weaning and recovery: After stable rewarming was achieved, CPB was discontinued. Postoperative ventilator support was continued for 30 min. The ventilator settings (respiratory rate: 60–70 breaths/min; tidal volume: 8–10 mL/kg) were adjusted according to arterial blood gas analyses.
5. Control group and postoperative care: Sham-operated control animals underwent identical surgical procedures, including intubation and vascular cannulation, but were not subjected to CPB or DHCA. For postoperative analgesia, all animals received standardized intramuscular injections of acetaminophen.

Twenty-one rats were subjected to the CPB/DHCA procedure. Twelve animals completed the full experimental protocol and were included in the final analysis. Nine rats were excluded: four because of technical failure during CPB establishment, two because of acute complications (e.g., air embolism), and three because of death within 24 h post-operation. At 24 h postmodelling, the rats were euthanized via pentobarbital sodium (50 mg/kg) for tissue harvesting. Upon harvest, target tissues were immediately snap-frozen in liquid nitrogen and stored at −80 °C for subsequent analysis. The hippocampus was then processed for transcriptomic profiling using RNA sequencing. Owing to its recognized susceptibility to ischemia, the hippocampus was chosen for molecular analysis as it is a sensitive indicator of global cerebral injury after DHCA (13, 19).

### RNA extraction, sequencing, and bioinformatic analysis

Total RNA was extracted using TRIzol (Invitrogen). The RNA concentration and purity were determined by measuring the A260/A280 ratio using a NanoDrop ND-1000 (Thermo Fisher Scientific, Waltham, MA, USA). RNA samples were immediately frozen and stored at −80 °C until use. The miRNA was sequenced by CloudSeq, Inc. (Shanghai, China). Small RNA libraries were constructed using a GenSeq® Small RNA Library Prep Kit (GenSeq, Inc.) following the manufacturer's instructions. Briefly, 3′ and 5′ adapters were subsequently ligated to the RNA samples. Subsequently, the adapter-ligated RNA was reverse transcribed into cDNA, followed by polymerase chain reaction (PCR) amplification. After amplification, miRNA fractions were selected from the cDNA libraries based on size and then sequenced on a platform.

After sequencing, the raw data were subjected to image analysis, base calling, and initial quality filtering. Quality was first controlled using the Q30 standard. Adapter sequences were removed using cutadapt (v1.9.3), retaining reads ≥15 nt after trimming. These reads were subsequently aligned to a combined pre-miRNA reference (including known miRBase entries and newly predicted pre-miRNAs) via Novoalign (v3.02.12), permitting up to one mismatch. Mature miRNA-mapped reads were counted as raw expression values and normalized using TPM (tags per million aligned miRNAs). Novel miRNAs were predicted using miRDeep2 (v2.0.0.5) on pooled trimmed reads from all the samples. miRNAs with an absolute fold change ≥1.5 and a *P* value ≤0.05 were considered as differentially expressed. Experimentally supported or predicted miRNA targets were identified using established tools, and miRNA‒target networks were visualized with Cytoscape (v2.8.0). A functional enrichment analysis was performed on the target genes of the top 20 differentially expressed miRNAs.

### Patients

One hundred forty consecutive patients undergoing open surgical repair for AAD who were admitted to cardiac surgery center from January 2024 to June 2024 were enrolled. This retrospective study was reviewed and approved by the Fujian Medical University Union Hospital Ethics Committee and strictly complied with the Declaration of Helsinki. Informed consent was waived by the ethics committee because of the retrospective and anonymized nature of the data. The inclusion criteria were as follows: (1) age ≥18 years and (2) underwent open surgical repair. The exclusion criteria were as follows: (1) pre-existing neurological or psychiatric disorders (e.g., dementia, stroke, schizophrenia, or depression); (2) concurrent chronic hepatic or renal dysfunction; (3) preoperative shock or haemodynamic instability secondary to cardiac tamponade; and (4) death within 24 h after surgery. Eligible patients were divided into two groups: patients with PONC and patients without PONC. The diagnosis of stroke was made clinically and confirmed by computed tomography or magnetic resonance imaging. Neurological deficit severity was assessed using the National Institutes of Health Stroke Scale at admission. Postoperative delirium was assessed using the Richmond Agitation–Sedation Scale and Confusion Assessment Method for the ICU, while the Glasgow Coma Scale provided objective criteria for defining postsurgical coma.

### Blood collection

Venous blood samples (5 mL) were collected from all participants using EDTA-coated tubes at two time points: pre-anaesthesia induction and 24 h postoperatively. Plasma was isolated by low-speed centrifugation (1,500×g, 4 °C, 15 min), after which the supernatant was aliquoted into cryovials and stored at −80 °C for subsequent biochemical assays.

### Surgical procedure

The operation was performed under general anaesthesia via a sternal incision. General anaesthesia was induced with intravenous midazolam, sufentanil, and etomidate and maintained with sevoflurane and a continuous infusion of sufentanil and propofol. Muscle relaxation was achieved with rocuronium. During CPB, anaesthesia was maintained with propofol and midazolam. Haemodynamic monitoring included intra-arterial pressure and transesophageal echocardiography. After heparinization, CPB was established by venous and arterial cannulation. When the nasopharyngeal temperature had decreased to 32 °C, the ascending aorta was clamped, and 4 °C cold blood cardioplegia was infused for myocardial protection. Subsequently, the repair operation was performed. After cooling to the required temperature (nasopharyngeal temperature, 20–23 °C; rectal temperature, 23–26 °C), circulatory arrest began, and bilateral selective antegrade cerebral perfusion was performed. The flow rate was approximately 8–10 mL/kg/min. For arch repair, total arch replacement using a 4-branched Dacron graft with stented elephant trunk implantation or open triple-branched stent graft placement was performed, as previously reported (20, 21). The triple-branched stent graft technique was selected when preoperative CT measurements revealed that the diameters of the native aortic arch and arch vessels were 10%–20% smaller than those of the graft, and the distances between adjacent arch vessels matched the corresponding interbranch distances of the stent graft. Otherwise, total arch replacement with a 4-branched Dacron graft and a stented elephant trunk was performed.

### miRNA extraction, cDNA synthesis, and qPCR quantification

For miRNA expression profiling, total RNA was isolated using the miRcute miRNA Isolation Kit (TIANGEN, Beijing, China; DP503), followed by first-strand cDNA synthesis using the miRcute Plus miRNA First-Strand cDNA Synthesis Kit (TIANGEN Biotech, Beijing, China; KR211-02), in strict accordance with the manufacturer's protocols. Quantitative PCR analysis was performed using the miRcute Plus miRNA SYBR Green qPCR Kit (TIANGEN Biotech, Cat# FP411-02) in strict compliance with standardized protocols. Serum miR-29 expression was quantified through by real-time PCR, where threshold cycle (Ct) values derived from RT‒PCR amplification kinetics were computationally transformed using the 2^−ΔΔCt^ algorithm. The relative expression metric was defined as ΔΔCt = [Ct (target miRNA) − Ct (U6 snRNA)] − [Ct (calibrator sample) − Ct (U6 snRNA)], with U6 snRNA serving as the endogenous normalization control. The miRNA primers used (Sunya Biotech) were as follows: miR-29a-5p forward, 5′-CCGCGACTGATTTCTTTTGGTGTTCAG-3′, and miR-29b-3p forward, 5′-CCGCTAGCACCATTTGAAATCAGTGTT-3′. The sequences were designed by Sunya Biotech (ISO-certified) following stem‒loop RT‒qPCR design principles.

### Data analysis and statistics

Statistical analyses were performed using SPSS (version 20.0; IBM, USA). Normally distributed measurement data are expressed as the mean ± standard deviation, whereas non-normally distributed are expressed as the median and interquartile range. Two independent groups were compared using t tests or Mann‒Whitney *U* tests (for continuous variables) and chi‒square tests or Fisher's exact tests (for categorical variables). Significant univariate predictors (*p* < 0.05) were advanced to multivariate logistic regression for identifying independent PONC risk factors, and a nomogram was constructed. The prediction model was evaluated through internal validation using receiver operating characteristic (ROC) curves, calibration curves, decision curve analysis (DCA), and confusion matrices. Survival probabilities over the 30-day observation period were estimated using the Kaplan‒Meier method, with between-group comparisons assessed via log-rank test. A two-tailed *p*-value < 0.05 was considered statistically significant.

## Results

### Differential gene expression in the hippocampus of rats during CPB/DHCA

**Figure 2:**
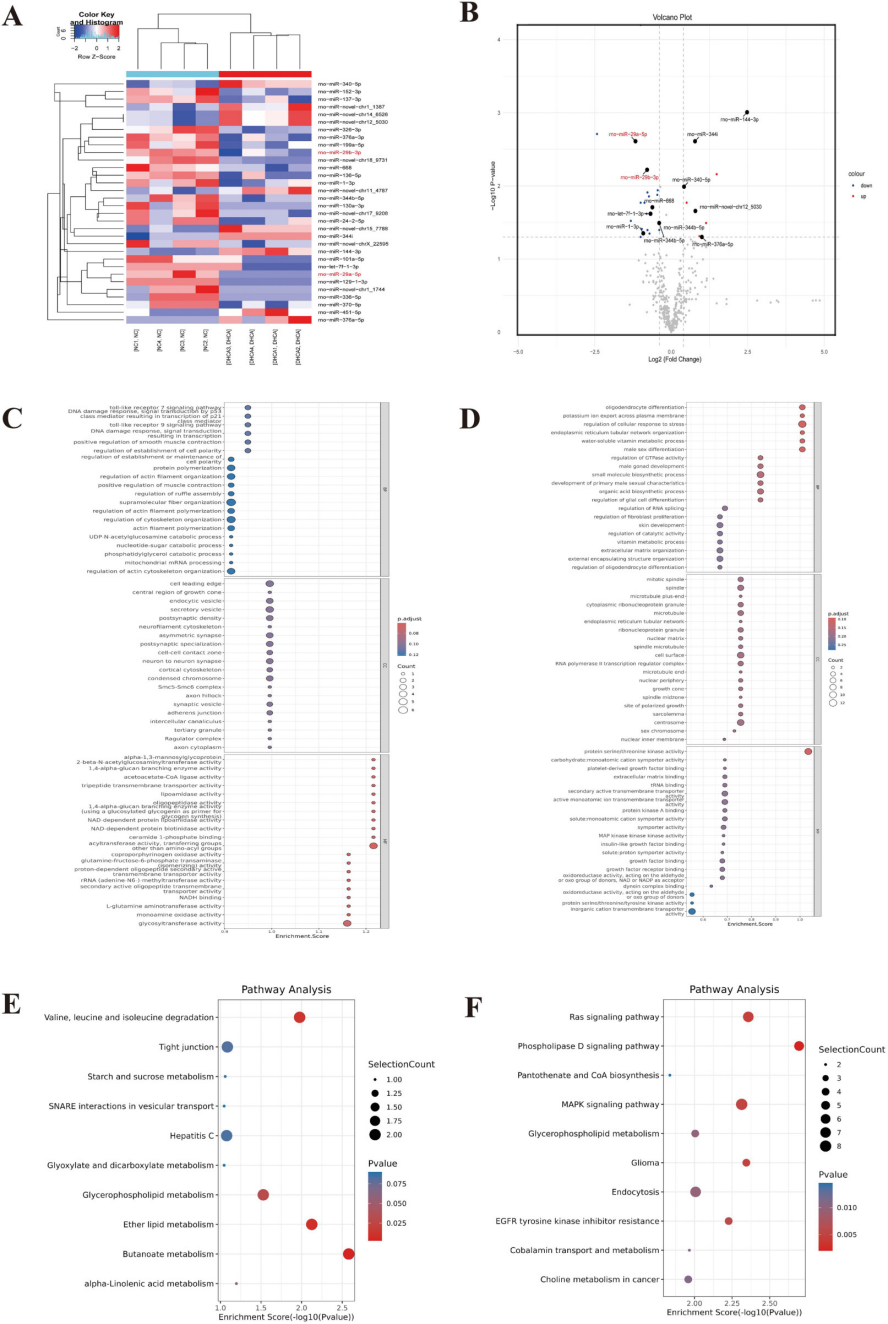
Hippocampal RNA-Seq analysis of differential gene expression between the CPB/DHCA group (*n* = 4) and the control group (*n* = 4). **(A)** Profiling heatmap of upregulated and downregulated miRNAs between the CPB/DHCA and control groups. **(B)** Volcano plot of DEGs between the CPB/DHCA and control groups. **(C)** GO enrichment analysis of upregulated genes (BP, MF, and CC) at level 2. **(D)** GO enrichment analysis of downregulated genes (BP, MF, and CC) at level 2. **(E)** KEGG pathway enrichment of upregulated genes between the CPB/DHCA and control groups. **(F)** KEGG pathway enrichment of downregulated genes between the CPB/DHCA and control groups. DHCA, deep hypothermic circulatory arrest; CPB, cardiopulmonary bypass; DEGs, differentially expressed genes; BP, biological process; MF, molecular function; CC, cellular component; KEGG, Kyoto encyclopedia of genes and genomes.

To investigate miRNA dynamics under hypothermic circulatory arrest conditions, high-throughput sequencing coupled with comparative transcriptomic analysis of hippocampal tissues from the CPB/DHCA group and control group was used to characterize endogenous miRNA profiles. More than six hundred miRNAs were identified by high-throughput sequencing. Differentially expressed miRNAs were defined by a selection criterion of |FC|>1.5 with statistical significance (*P* < 0.05). Through this analytical pipeline, 31 miRNAs met the defined thresholds, comprising 10 upregulated and 21 downregulated miRNAs (Figure 2A). Notably, the changes in the expression of miR-29a-5p and miR-29b-3p were the most pronounced (Figure 2B).

Subsequent functional annotation was performed through integrated Gene Ontology (GO) and Kyoto Encyclopedia of Genes and Genomes (KEGG) pathway enrichment analyses to systematically characterize miRNA-mediated regulatory networks. Functional annotation analysis revealed the GO landscape across biological processes (BP), molecular functions (MF), and cellular components (CC), as depicted in Figures 2C,D. KEGG pathway profiling revealed predominant enrichment of differentially expressed genes (DEGs) in three critical pathways: MAPK signalling, tight junction, and gap junction assembly (Figures 2E,F). These pathways are functionally implicated in BBB homeostasis through coordinated regulation of endothelial cell–cell adhesion complexes and paracellular permeability (22, 23).

### Patient demographic and clinical characteristics

Between January and June 2024, 140 consecutive patients with AAD were initially enrolled in this study. Following rigorous screening, 45 patients were excluded based on the following predefined criteria: incomplete clinical records (*n* = 23), preexisting neurological/psychiatric comorbidities (*n* = 7), chronic hepatorenal insufficiency (*n* = 8), and mortality events (aortic rupture-related deaths, *n* = 4; death within 24 h after surgery, *n* = 3). Ultimately, 95 eligible patients composed the final analytical cohort. The baseline demographic and clinical characteristics of the study cohort are summarized in Table 1.

**Table 1:** The demographic and preoperative data of patients.

Variables	Total (*n* = 95)	Non-PONC (*n* = 70)	PONC (*n* = 25)	*P*
Age (years)	54.00 (44.50, 63.50)	53.50 (44.00, 63.00)	58.00 (51.00, 64.00)	0.314
Male	71 (74.74)	53 (75.71)	18 (72.00)	0.714
Body mass index (kg/m2)	24.40 (22.00, 26.70)	23.85 (21.65, 26.10)	25.40 (22.90, 28.70)	0.088
Smoking	35 (36.84)	29 (41.43)	6 (24.00)	0.121
Hypertension	66 (69.47)	49 (70.00)	17 (68.00)	0.852
Diabetes mellitus	3 (3.16)	2 (2.86)	1 (4.00)	1
Ejection fraction (%)	65.00 (61.95, 68.20)	65.00 (61.93, 68.08)	65.20 (62.10, 68.20)	0.685

Continuous variables are presented as median (interquartile range); categorical variables are presented as *n* (%). PONC, postoperative neurological complications.

**Figure 3:**
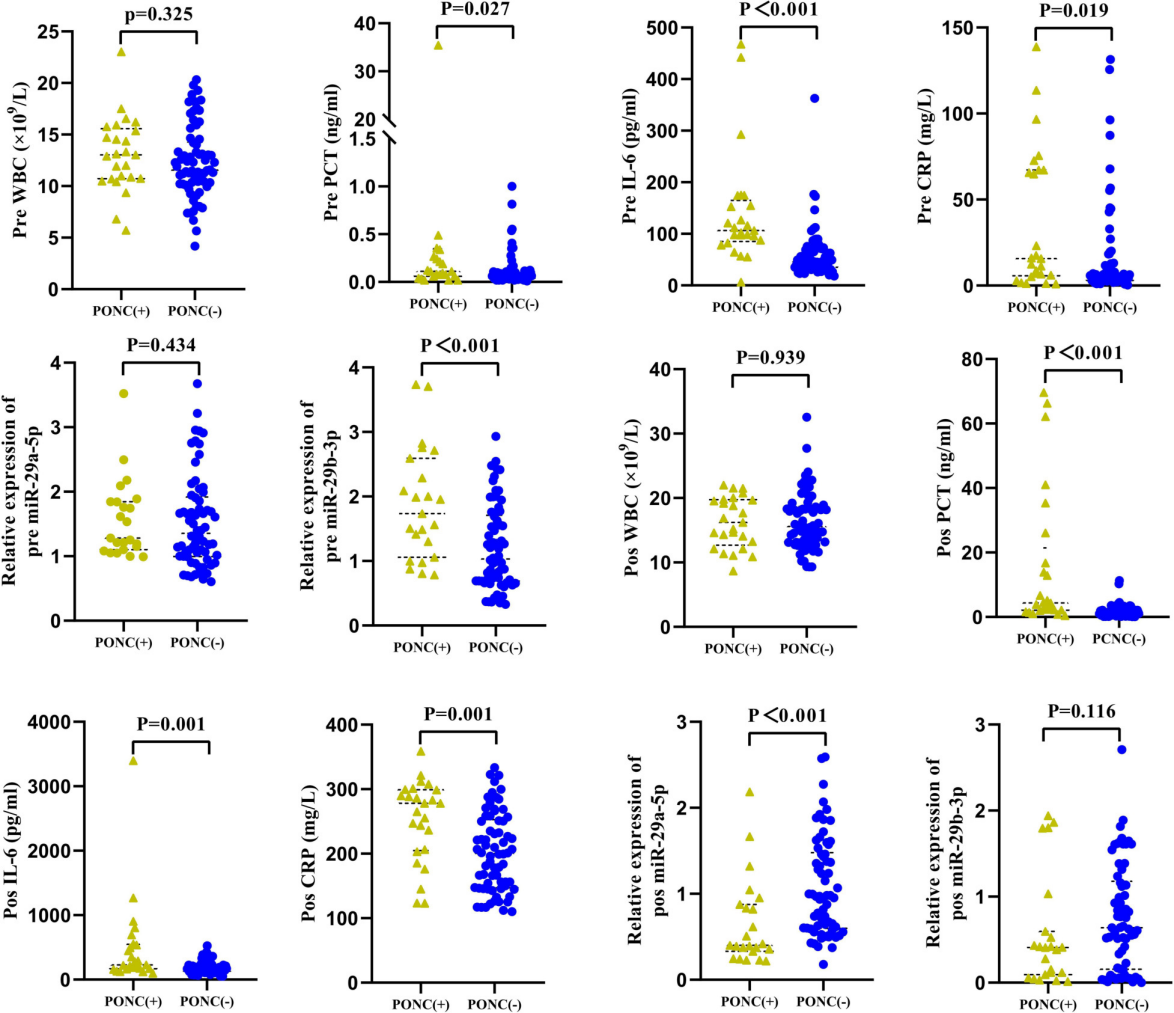
Comparison of preoperative and postoperative laboratory examinations between the two groups. WBC, white blood cell; CRP, C-reactive protein; PCT, procalcitonin; IL-6, interleukin-6.

A comparative analysis of the perioperative parameters revealed that compared with the non-PONC cohort, the PONC cohort had significantly longer CPB times, extended aortic cross-clamp durations, longer mechanical ventilation requirements, and longer ICU stays (Table 2). Furthermore, in-hospital mortality rates were significantly higher in the PONC cohort (28% vs. 4.29%, *p* = 0.003). Preoperative biomarker analysis revealed significant intergroup disparities (Figure 3). Compared with non-PONC controls, the PONC cohort demonstrated marked increases in the expression of inflammatory mediators, such as CRP (*p* = 0.019), procalcitonin (PCT) (*p* = 0.027), IL-6 (*p* < 0.001), and miR-29b-3p (*p* < 0.001). Postoperative inflammatory marker levels also demonstrated similar trends (Figure 3). CRP (*p* = 0.001), PCT (*p* < 0.001), and IL-6 (*p* = 0.001) levels significantly increased in the PONC group. Conversely, miR-29a-5p expression was markedly decreased in PONC patients (*p* < 0.001).

**Table 2:** Perioperative and postoperative data.

Variables	Total (*n* = 95)	Non-PONC (*n* = 70)	PONC (*n* = 25)	*P*
CPB duration (min)	142.00 (123.50, 167.00)	140.00 (120.25, 161.00)	164.00 (137.00, 193.00)	0.012
Aortic cross clamp duration (min)	64.00 (48.00, 87.50)	61.00 (47.250, 83.00)	86.00 (60.00, 93.00)	0.017
SCP duration (min)	14.00 (10.00, 20.00)	13.25 (10.00, 19.75)	16.00 (14.00, 32.00)	0.060
MHCA duration (min)	2.00 (0.00, 3.00)	2.00 (0.00, 3.00)	2.00 (0.00, 4.00)	0.656
MNT (°C)	22.50 (22.00, 23.00)	22.50 (22.00, 23.00)	22.50 (22.00, 23.50)	0.766
MRT (°C)	25.00 (23.50, 25.50)	25.00 (23.50, 25.88)	25.00 (23.60, 25.10)	0.777
Ventilation support duration (h)	40.00 (20.00, 83.00)	24.50 (18.00, 60.00)	103.00 (51.00, 160.00)	<.001
ICU stay (min)	86.00 (50.50, 155.50)	64.50 (44.25, 120.00)	166.00 (109.00, 232.00)	<.001
Length of hospital stay (d)	10.00 (9.00, 15.00)	10.00 (9.00, 15.00)	9.50 (8.00, 13.00)	0.673
In-hospital mortality	10 (10.53)	3 (4.29)	7 (28.00)	0.003

Continuous variables are presented as median (interquartile range); categorical variables are presented as *n* (%). PONC, postoperative neurological complications; CPB, cardiopulmonary bypass; min, minutes; SCP, selective cerebral perfusion; MHCA, moderate hypothermic circulatory arrest; MNT, minimum nasopharyngeal temperature; MRT, minimum rectal temperature.

### Univariate and multivariate logistic regression analyses

Univariate logistic regression revealed multiple perioperative predictors of PONC, including preoperative inflammatory biomarkers, preoperative miR-29b-3p expression, postoperative miR-29a-5p expression, and CPB time. Notably, multivariate adjustment confirmed that preoperative miR-29b-3p expression (OR = 2.53, 95%CI 1.17–5.47, *p* = 0.018) and postoperative miR-29a-5p expression (OR = 0.21, 95%CI 0.05–0.96, *p* = 0.044) were independent risk factors for PONC development, whereas IL-6, CRP, and CPB time lost statistical significance in the multivariable model (Table 3).

**Table 3:** Univariate and multivariate analyses of all patients.

Variables	Univariate analysis		Multivariate analysis	
	OR (95%CI)	*P* value	OR (95%CI)	*P* value
Male	0.82 (0.29–2.31)	0.714		
Age (years)	1.02 (0.98–1.06)	0.267		
Body mass index (kg/m^2^)	1.11 (0.98–1.26)	0.114		
Hypertension	0.91 (0.34–2.44)	0.852		
Diabetes mellitus	1.42 (0.12–16.34)	0.780		
Smoking	0.45 (0.16–1.26)	0.126		
Pre IL-6 (pg/mL)	1.02 (1.01–1.03)	<.001	1.01 (1.00∼1.02)	0.054
Pre CRP (mg/L)	1.02 (1.01–1.03)	0.016	1.01 (0.99∼1.02)	0.543
CPB duration (min)	1.01 (1.01–1.02)	0.035	1.00 (0.99∼1.01)	0.526
SCP duration (min)	1.03 (0.99–1.07)	0.176		
Pre miR-29a-5p	1.08 (0.55–2.14)	0.820		
Pre miR-29b-3p	3.13 (1.57–6.26)	0.001	2.53 (1.17∼5.47)	0.018
Post miR-29a-5p	0.17 (0.05–0.58)	0.005	0.21 (0.05∼0.96)	0.044
Post miR-29b-3p	0.58 (0.25–1.33)	0.195		

CRP, c-reactive protein; IL-6, interleukin-6; CPB, cardiopulmonary bypass; SCP, selective cerebral perfusion; pre miR-29a-5p, preoperative miR-29a-5p; pre miR-29b-3p, preoperative miR-29b-3p; post miR-29a-5p, postoperative miR-29a-5p; post miR-29b-3p, postoperative miR-29b-3p.

*P* value less than 0.05, indicating a statistically significant difference between groups.

### Development and validation of a nomogram prediction model

**Figure 4:**
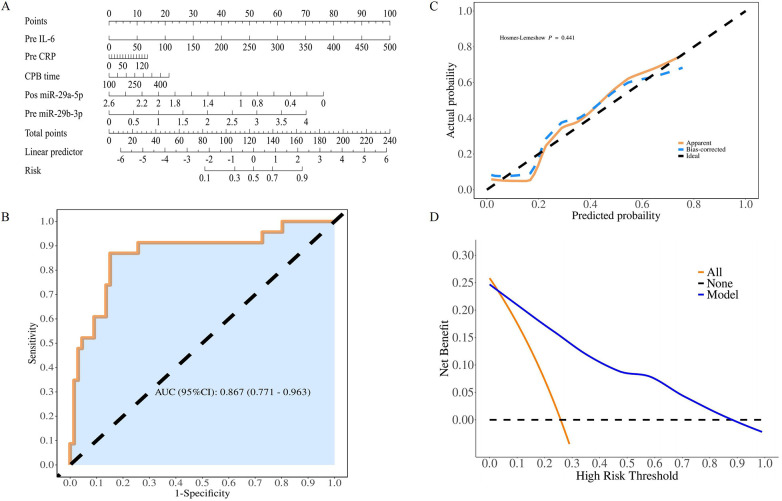
Development and validation of a nomogram prediction model. **(A)** Nomogram for predicting postoperative neurological complications. The nomogram model assigns a score on a 0–100-point scale according to the regression coefficient, and the total score for a patient can be calculated by summing the scores of each variable. **(B)** Receiver operating characteristic curve evaluation. **(C)** Hosmer–Lemeshow calibration curve. **(D)** The decision curve analysis curve for the validation sets suggested that the maximum net benefit of 0.23 was achieved when the threshold value was between 0.3 and 0.87. IL-6, interleukin-6; CRP, c-reactive protein; CPB, cardiopulmonary bypass; premiR-29b-3p, preoperative miR-29b-3p; and postmiR-29a-5p, postoperative miR-29a-5p.

A clinically oriented nomogram incorporating the identified independent predictors was developed (Figure 4A), followed by rigorous multidimensional validation. The nomogram exhibited strong discriminative ability, with an area under the ROC curve (AUC) of 0.867 (Figure 4B). At the optimal probability threshold, the model achieved high overall accuracy, sensitivity, and specificity, along with favorable predictive values and likelihood ratios. The diagnostic odds ratio and Youden index further supported its excellent diagnostic performance (Table 4). The model exhibited optimal calibration fidelity, as evidenced by the nonsignificant Hosmer–Lemeshow test results (*p* = 0.441) (Figure 4C). Decision curve analysis revealed substantial clinical utility across the 3%–87% risk threshold spectrum, achieving peak net benefit (Figure 4D).

**Table 4:** Model confusion matrix.

AUC	Accuracy	Sensitivity	Specificity	PPV	NPV	LR+	LR-	DOR	Youden Index	cut off
(95% CI)	(95% CI)	(95% CI)	(95% CI)	(95% CI)	(95% CI)	(95% CI)	(95% CI)	(95% CI)	(95% CI)	
0.867	0.854	0.848	0.87	0.949	0.667	6.52	0.17	38.35	0.718	0.307
(0.771–0.963)	(0.763–0.920)	(0.762– 0.935)	(0.732– 1.000)	(0.893– 1.000)	(0.498–0.835)	(2.470–17.230)	(0.070–0.430)	(9.870–148.970)	(0.494–0.935)	

PPV, positive predictive value; NPV, negative predictive value; LR+, positive likelihood ratio; LR-, negative likelihood ratio; DOR, diagnostic odds ratio.

### Survival curve

**Figure 5:**
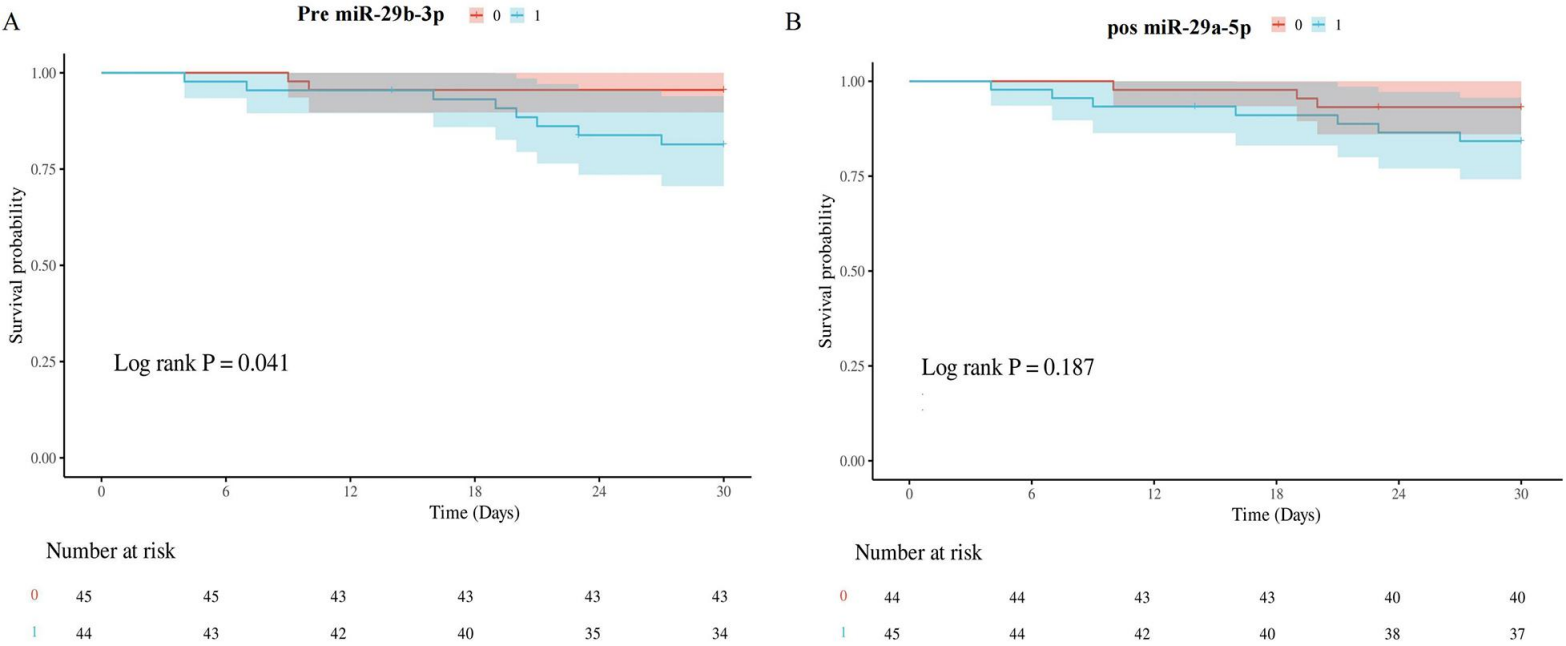
Survival curve. **(A)** Kaplan–Meier analysis stratified by median premiR-29b-3p expression thresholds revealed significant survival discrimination (log-rank *P* = 0.041), with an elevated hazard ratio (HR = 4.372, 95%CI 0.928–20.596). **(B)** Kaplan–Meier analysis stratified by median postmiR-29a-5p expression thresholds revealed no significant difference in prognosis [log-rank *P* = 0.187; hazard ratio (HR) = 2.415; 95%CI 0.624–9.339].

Thirty-day survival analyses stratified by miRNA expression thresholds (median premiR-29b-3p and postmiR-29a-5p levels) revealed distinct prognostic patterns. Preoperative miR-29b-3p expression was significantly associated with survival (log-rank *P* = 0.041; Figure 5A), with an elevated hazard ratio (HR = 4.372; 95%CI 0.928–20.596). In contrast, postoperative miR-29a-5p expression was not significantly associated with prognosis, indicating only a marginal increase in risk (Figure 5B).

## Discussion

Circulating miRNAs are known for their stability and have emerged as promising diagnostic tools for central nervous system injury. However, their reliability in predicting or diagnosing postoperative brain injury following AAD surgery remains to be validated. Our study highlights the critical role of miRNAs in regulating diverse cerebral pathological processes and identifies them as novel biomarkers. Notably, cross-species analyses revealed differential postoperative miR-29 fluctuations across neural and circulatory compartments, establishing their association with PONC in AAD patients. These findings not only identify miR-29 as a potential biomarker for DHCA-associated neurological damage but also elucidate its mechanistic involvement in PONC, shedding light on previously unexplored molecular pathways.

This study provides the first evidence of decreased miR-29a-5p expression in the blood of patients who underwent DHCA. Notably, these findings were corroborated in animal models subjected to DHCA, in which miR-29a-5p downregulation mirrored clinical observations, further confirming its consistent association with DHCA-induced physiological stress. The miR-29 family is widely recognized to play roles in neuroprotection, regulation of apoptosis, inflammation, and synaptic plasticity (24). In animal models, DHCA-induced cerebral ischemia‒reperfusion injury likely disrupts miR-29 expression, as observed in brain transcriptomic analysis. Previous studies have shown that miR-29 suppresses the expression of pro-apoptotic proteins and maintains blood–brain barrier integrity by targeting matrix metalloproteinases (25). Its downregulation during DHCA may exacerbate neuronal apoptosis, disrupt neurovascular coupling, and amplify inflammatory cascades, contributing to cognitive decline. Clinically, the parallel reduction in plasma miR-29a-5p levels in AAD patients with PONC suggests its systemic release from damaged neural tissue or impaired synthesis due to ischemic stress. This finding aligns with evidence linking circulating miR-29 to neurodegenerative conditions, in which its loss is correlated with synaptic dysfunction and memory deficits (26).

Mechanistically, our findings align with the pleiotropic role of miR-29b-3p in neural injury pathways. The preoperative elevation of miR-29b-3p emerged as an independent risk factor for PONC, which is consistent with its established capacity to exacerbate ischemic neuronal damage through apoptosis potentiation (via Bcl2L2 suppression) and redox imbalance (27, 28). Notably, miR-29b-3p antagomir administration demonstrated therapeutic potential in preclinical models, attenuating oxidative stress and restoring endothelial homeostasis—a critical pathway for vascular repair in aortic dissection (29). Cross-disease analyses further reinforce its pathological significance, with upregulated miR-29b-3p being implicated in moyamoya disease leukocytes (30) and cancer progression (31).

Notably, the expression patterns of miR-29b-3p and miR-29a-5p differed across the experimental and clinical settings. While both were downregulated post-DHCA in rats, preoperative plasma miR-29b-3p (but not miR-29a-5p) was elevated in PONC patients, with no postoperative differences observed. Despite sharing neuroprotective functions through apoptosis inhibition and oxidative stress mitigation (24, 27), their compartment-specific dynamics may reflect distinct biological roles. Nucleus-localized miR-29b-3p (vs. cytoplasmic miR-29a) demonstrates limited peripheral release efficiency, which is supported by its 10–20-fold lower serum levels than those of cytoplasmic miRNAs (e.g., miR-122 in hepatocellular carcinoma), likely due to cellular barrier constraints and prolonged nuclear retention (32, 33).

The association between the level of miR-29 and PONC underscores its potential as a prognostic biomarker. DHCA, while necessary for aortic repair, induces cerebral hypoperfusion and oxidative stress, triggering miR-29-dependent pathways that may fail to counteract neuronal damage. Notably, miR-29 is also involved in mitigating endothelial dysfunction and amyloid-beta accumulation (34, 35), both of which are implicated in postoperative delirium and dementia. Our findings suggest that miR-29a-5p depletion post-DHCA could reflect a compromised adaptive response to ischemic insults, rendering patients vulnerable to cognitive impairment. Preclinical evidence substantiates this hypothesis, demonstrating that miR-29 expression is correlated with neuronal survival in ischemic brain injury, while preoperative miR-29b-3p elevation independently predicts 30-day postoperative mortality.

### Limitations

While this study established a significant correlation between miR-29 dysregulation and PONC, several limitations warranted consideration. First, this study was unable to establish a direct causal relationship between miR-29 levels and the pathogenesis of PONC. Second, our mechanistic exploration was confined to transcriptomic analysis of the rat hippocampus. Third, the study lacked corroborative RNA-seq data from patient blood or tissue samples, which limited our ability to directly translate the rodent transcriptomic findings to the human pathophysiology. Finally, the absence of functional validation through *in vitro* neuronal models or *in vivo* behavioral assays in animals meant the precise neuroprotective or detrimental roles of miR-29 in the context of DHCA remains to be experimentally confirmed.

## Future directions

To address the aforementioned limitations and advance the clinical translation of our findings, future research should prioritize the following directions. First, conducting *in vitro* and *in vivo* experiments is essential to definitively establish the causal roles of miR-29 in the pathogenesis of PONC and to elucidate their precise molecular targets. Second, building on current hippocampal data, subsequent animal studies should analyze miRNA expression in other cognition-critical brain regions and at multiple time points after DHCA to delineate the spatiotemporal dynamics of injury and repair. Finally, prospective, multicenter, longitudinal studies are needed to validate the predictive accuracy of the miR-29 based nomogram, while parallel profiling of circulating miRNAs correlated with patient RNA-seq data will help bridge the translational gap between rodent models and human disease.

## Conclusion

In conclusion, this study identified dysregulated miR-29 expression as a key molecular alteration associated with PONC after CPB/DHCA. Furthermore, we validated circulating miR-29b-3p as an independent predictor of PONC and mortality in patients with AAD. These findings provide a basis for the early risk assessment of cerebral injury in this high-risk surgical population.

## Data Availability

The datasets presented in this study can be found in online repositories. The datasets are available in the GEO repository (GSE 298179).
